# Novel Regulators of the IGF System in Cancer

**DOI:** 10.3390/biom11020273

**Published:** 2021-02-12

**Authors:** Caterina Mancarella, Andrea Morrione, Katia Scotlandi

**Affiliations:** 1 IRCCS Istituto Ortopedico Rizzoli, Laboratory of Experimental Oncology, 40136 Bologna, Italy; caterina.mancarella@ior.it; 2 Department of Biology, Sbarro Institute for Cancer Research and Molecular Medicine and Center for Biotechnology, College of Science and Technology, Temple University, Philadelphia, PA 19122, USA; Andrea.Morrione@temple.edu

**Keywords:** IGF system, cancer, transcriptional regulators, functional regulation, circular RNAs, IGF2BPs, ADAR, DDR1, E-cadherin, decorin

## Abstract

The insulin-like growth factor (IGF) system is a dynamic network of proteins, which includes cognate ligands, membrane receptors, ligand binding proteins and functional downstream effectors. It plays a critical role in regulating several important physiological processes including cell growth, metabolism and differentiation. Importantly, alterations in expression levels or activation of components of the IGF network are implicated in many pathological conditions including diabetes, obesity and cancer initiation and progression. In this review we will initially cover some general aspects of IGF action and regulation in cancer and then focus in particular on the role of transcriptional regulators and novel interacting proteins, which functionally contribute in fine tuning IGF1R signaling in several cancer models. A deeper understanding of the biological relevance of this network of IGF1R modulators might provide novel therapeutic opportunities to block this system in neoplasia.

## 1. Introduction

The insulin-like growth factor (IGF) system is a network of ligands, binding proteins and receptors regulating crucial physiological and pathological biological processes. The system includes three major ligands (IGF1, IGF2 and insulin), four membrane receptors (the insulin receptor (IR), the IGF1R, the mannose 6-phosphate receptor (M6P/IGF2R) and the insulin receptor-related receptor (IRR)), and at least six circulating IGF-binding proteins (IGFBPs) [1,2,3,4]. In physiology, the IGF axis drives not only long-term effects like growth, development, differentiation and maintenance of cell functions but also short-term effects including glucose and amino-acid uptake, glucose utilization, lipids and proteins metabolism [1,3,5,6]. A correct and fine-tuned regulation of the IGF axis is necessary for health during lifespan. Alterations in IGF expression or function result in pathological conditions including diabetes, growth retardation, osteoporosis, neurodegenerative diseases, obesity and cancer [1,7,8]. The variety of physiological and pathological responses elicited by the IGF axis inside the cell is not reflected in the relatively small numbers of components that are canonically recognized as part of this system. The biological action of IGFs can be modulated by a variety of hormones and growth factors meanwhile the IGFs can enhance signals driven by other factors [6,9] suggesting that additional studies are still required to fully understand the mechanisms of IGF system action and regulation [9]. This review will discuss IGFs action in cancer particularly focusing on how novel regulators integrate the IGF system and how these biological networks affect tumor development and progression, response to therapies and metabolism and might provide novel therapeutic opportunities to block this system.

## 2. The IGF System: Receptors, Ligands and Binding Proteins

Bioactivity of the IGF system initiates with ligands (IGFs and insulin) binding to their cognate receptors. The two major receptors of the IGF system are the IGF1R and IR. They are transmembrane tyrosine kinase receptors (RTKs) composed of two α and two β subunits involved in ligand binding and signal transduction, respectively [10]. The IGF1R is expressed in a variety of human tissues and its activation results in the regulation of cell survival, proliferation, differentiation and protein synthesis [9].

The IR exists in two splicing isoforms: the IR-A, which is mainly expressed in fetal tissues and cancer cells, driving mitogenic signals and partially overlapping with IGF1R signaling [11], and the IR-B, which is preferentially expressed in adult tissues (for a detailed review please see [5]), and mediates metabolic functions [5]. The high sequence homology (60%) and the frequent co-expression of the IGF1R and IR determine the formation of hybrid receptors, consisting of an insulin αβ hemi-receptor and an IGF1 αβ hemi-receptor [12]. Interestingly, IR-A and IR-B are equally capable of forming hybrid receptors with the IGF1R, but those hybrids are functionally distinct [12].

Different downstream responses elicited by the receptors are strictly connected to the different expression pattern and ligand affinity. There are major differences between ligands properties and receptors affinities, which have been recently discussed [3,5]. Briefly, insulin displays the highest affinity for the IR >> IR/IGF1R hybrid > IGF1R; IGF1 displays the highest affinity for IGF1R > IR/IGF1R hybrid > IR; IGF2 displays the highest affinity to IGF1R/IGF2R > IR-A > IR/IGF1R hybrid >> IR-B [3].

While IGF2R lacks intrinsic kinase activity and acts as a scavenger of extracellular IGF2 [13], the IRR has been long viewed as an orphan receptor but recent data have demonstrated that the IRR acts as an extracellular Alkali Sensor [2,14].

A critical modulator of IGF bioactivity is the superfamily of 6 circulating IGFBPs (IGFBP1-6). IGFBPs are characterized by high affinity for IGF1 and IGF2 but not for insulin. Accordingly, 99% of circulating IGFs exist in complex with IGFBPs [15]. Thus, IGFBPs elicit a major modulatory role on IGF-dependent function by prolonging the half-life of IGFs, regulating the clearance of the IGFs, providing tissue specific localization and regulating binding to their receptors [8,15]. IGFBPs also elicit IGF-independent functions interacting with a variety of non-IGF binding partners at the cell surface and within the cell, in both the cytoplasm and the nucleus, thus modulating proliferation and migration [16]. The combined activity of binding proteins, ligands and receptors contributes to the activation of intracellular signaling pathways. Upon ligand binding, the kinase domain of the IGF1R, IR or hybrid receptors undergoes auto-transphosphorylation on tyrosine residues in the tyrosine-kinase domain and receptor activation with subsequent recruitment of downstream effector proteins including the insulin receptor substrate (IRS) proteins IRS-1-6 and Src homology 2 domain-containing transforming protein (Shc) [9]. Phosphorylation of these proteins leads to downstream activation of the phosphoinositide 3-kinase (PI3K)-Akt [17] and mitogen-activated protein kinase (MAPK) [18] pathways and the regulation of the aforementioned effects on metabolism and cell behavior.

## 3. The IGF System in Cancer

To date, the role of IGF system in cancer onset and progression has been documented in a variety of human malignancies [1,7,19]. Despite the vast majority of studies originally focused on the specific role of the IGF1R [20], it is now clear that expression or functional alterations of all components of the IGF axis are important factors in contributing to IGF1R action in cancer. Epidemiologic evidences indicated that high serum levels of IGF1 correlate with increased risk of cancer [21,22]. Accordingly, patients affected by Laron syndrome, a disease characterized by congenital deficiency of IGF1, do not develop cancer [23]. In 1993, Sell and colleagues demonstrated that the simian virus 40 large tumor antigen (SV40 TAg) was unable to transform fibroblasts derived from mouse embryos homozygous for a null mutation of the *igf1r* gene (R^−^ cells) [20]. Subsequent studies demonstrated that a variety of viral and cellular oncogenes require an active IGF1R for transformation [24] including the human papilloma virus E5 [25] and E7 protein [26], an activated c-Ha-*ras* oncogene [27], c-src [28], the Ewing sarcoma fusion protein EWS-ETS [29], the ETV6-NTRK3 chimeric tyrosine kinase [30], overexpressed growth factor receptors like EGFR [31], PDGFR [32] or IR [33]. In all cases, transformation of R^−^ cells was fully restored upon re-expression of the IGF1R [24].

### 3.1. Regulation of IGF System in Cancer

Dysregulation of the IGF axis strongly contributes to the malignant phenotype. Mechanisms correlating with an unbalanced IGF network include receptor overexpression, alterations in ligands availability and dysregulation of downstream signaling effectors, while mutations of the receptors are uncommon. A notable exception is represented by osteosarcoma, where recurrent mutations of IGF signaling genes have been recently uncovered [34]. These mutations include focal amplification of *IGF1R* and *IGF1,* and frameshift indels in the recessive cancer genes *IGF2R* and *IGFBP5* [34]. Amplification of the *IGF1R* was also identified in a percentage of breast tumors, co-occurring with the *CKS1BP7* pseudogene amplification [35], gastrointestinal stromal tumors [36], melanoma [37], and pancreatic adenocarcinoma [38]. Altered expression of members of the IGF axis is attributed to mutations or aberrant expression of transcriptional regulators. For instance, IGF1R activation occurs as a consequence of mutations of tumor suppressor genes [39,40] including breast cancer gene-1 (*BRCA1*) [41], the Wilm’s tumor protein-1 (*WT1*) [42], the von Hippel–Lindau gene (*VHL*) [43] and p53 *(TP53)* [44]. Interestingly, p53 regulates gene expression of other IGF system components including *INSR* [45], *IGF2* and *IGFBP3* [46]. Increased *INSR* expression in tumor cells is also modulated by the upregulation of Sp1 and HMGA1 transcription factors [47]. Several studies have identified proteins involved in alternative splicing that favor IR-A prevalence in tumor cells. As previously covered by Vella and colleagues, interesting correlations exist between IR-A abundance and CUG-BP1, hnRNP family, SR proteins and Muscleblind-like (MBNL) proteins [47]. Alterations in IGFs availability result in aberrant activation of the IGF axis. In this landscape, the overexpression of the pregnancy associated plasma protein-A (PAPP-A), a metalloproteinase that cleaves IGFBP4, increases the bioavailability of IGFs on the cell surface, which acts in autocrine/paracrine manner to increase locally available ligands for IGF1R and IR-A activation in tumor cells [48,49]. Similarly, the overexpression of the metalloproteinases ADAM17 and ADAM28, which specifically act on IGFBP3, favors cancer cells proliferation by enhancing IGF1 bioavailability [50,51]. In addition, loss-of-heterozygosity of *IGF2R* favors the interaction between IGF2 and IGF1R in different tumor types [52,53]. Finally, alterations in intracellular signaling molecules can alter IGF equilibrium. In glioma, a specific mutation in phosphatase and tensin homologue (*PTEN*) gene, a tumor suppressor and lipid phosphatase, determines the truncation of its C-terminal region with consequent gain of neo-morphic and phosphatase-independent activity, which stimulates IGF1 synthesis [54]. Among other downstream effectors, the docking protein IRS-1 is constitutively activated in a variety of solid tumors, including breast cancers, leiomyomas, Wilms’ tumors, rhabdomyosarcomas, liposarcomas, leiomyosarcomas and adrenal cortical carcinomas [55]. A recent pan-cancer study identified signaling via SHC family adapter proteins and PI3K/Akt/mTOR among the pathways highly mutated in cancer [56].

### 3.2. Effects of IGF System in Cancer Progression, Response to Therapies and Cellular Metabolism

Many previous reviews have nicely described the important role that the IGF system plays in transformation [7,57,58]. This section will highlight some examples of molecular mechanisms driven by IGF bioactivity, which modulate cell behavior, treatment response and metabolism.

The IGF axis drives cancer cell proliferation, cell–cell adhesion and migration. As mentioned above, the activation of the PI3K/Akt and MAPK pathways plays a critical role in mediating IGF action in cancer, and it is often associated with aberrant activation/inhibition of transcription factors. The PI3K/Akt pathway is the main regulator of the Forkhead box O (FoxO) family of transcription factors, which acts as a tumor suppressor in different tumors [59,60]. In thyroid cancer, IGF1-mediated activation of Akt promotes FoxO1 export from the nucleus, inhibition of FoxO1-mediated transcriptional activation of target genes like *CDKN1B* (p27KIP1) cell cycle inhibitor, thus promoting cell proliferation [61]. In addition, IGF1R activation determines phosphorylation and nuclear translocation of STAT3, which modulates transcriptional activation of cancer-associated genes. In ovarian cancer, the activation of the IGF1R/STAT3 axis promotes cell migration, invasion and in vitro spheroid formation and induces in vivo tumor growth [62]. Additional cancer genes modulated by a dysregulated IGF1R-STAT3 axis are *ALDH1* [63] and *Nanog* [64], which enhance the epithelial-to-mesenchymal transition (EMT)-associated cancer stem cells (CSC)-like properties in non-small cell lung cancer and colorectal cancer, respectively.

IGF1R signaling strongly associates with EMT [65]. The expression of EMT-associated proteins like N-cadherin, vimentin, Snail and Twist is positively associated with IGF1/IGF1R activation. However, the mechanisms characterizing this functional interaction are still not fully defined [66]. In hepatocellular carcinoma, EMT is driven by IGF1-induced activation of the transcription factor STAT5 [66], while in prostate cancer cells, IGF1 stimulation up-regulates ZEB1, a zinc finger homeodomain transcriptional repressor. ZEB1 increases the expression of mesenchymal markers such as fibronectin and N-cadherin while repressing E–cadherin, thus favoring cancer cell migration and invasion through the activation of the MAPK pathway [67]. Similarly, IGF1 enhances the expression of CYR61, a member of the extracellular matrix-associated CCN family, which triggers EMT specific features in vitro and in vivo [68,69]. Notably, in osteosarcoma and breast cancer, CYR61 controls the N-cadherin/E-cadherin ratio as well as the expression of other markers such as Snail, Slug, Vimentin, thereby favoring spheroid growth and cell invasion while impairing cell–cell and cell–matrix adhesion [68,69]. As recently reported, the IGF1/IGF1R axis promotes the activation of focal adhesion kinase (FAK) signaling, which in turn regulates nuclear accumulation of YAP (yes-associated protein/yes-related protein), a major component of the Hippo pathway, and increased expression of its target genes including *CYR61* [70].

Aberrant activation of IGF system in cancer has been associated with resistance to cytotoxic therapy, including chemotherapy and radiotherapy, and targeted therapy. IGF1R mediates resistance to cisplatin treatment in different tumor types [71,72]. Particularly, in vitro evidences derived from cisplatin-resistant cell lines derived from ovarian and testicular tumors indicated that IGF1R induction and activation of Akt downstream signaling represent major events in the acquisition of resistance. Accordingly, combination of anti-IGF agents with chemotherapeutics could promote re-sensitization to treatment in chemo-resistant disease [72]. In addition to the IGF1R, other effectors of the IGF system play a role in treatment response. In esophageal adenocarcinoma, treatment-resistant patients display high expression levels of IGFBP2 as compared to chemo-naive patients. Significantly, simultaneous IGFBP2 depletion and pharmacological inhibition of Akt and MAPK pathways sensitized esophageal adenocarcinoma cells to cisplatin therapy [73]. The relevance of IGFBPs in treatment response has been additionally reported in glioblastoma, where IGF2/IGF1R expression is associated with poor response to temozolomide. In addition, in vitro studies indicate a heterogeneous model where proliferation of temozolomide-resistant cells, characterized by an active IGF2/IGF1R axis, is controlled at paracrine level by temozolomide-sensitive cells, expressing high levels of circulating IGFBP6. Temozolomide treatment destroys treatment-responsive cells therefore enriching the tumor mass with resistant cells [74]. Recent evidence has highlighted the role that the IGF system plays in promoting resistance to novel epigenetic anti-cancer agents. In Ewing sarcoma cells, constitutive activation of the IGF1R confers resistance to inhibitors of the family of bromodomain and extra-terminal domain (BET) proteins, which recognize acetylated histone marks, thus recruiting supramolecular complexes to promote active transcription. Accordingly, over expression of a constitutively active form of Akt significantly increased resistance to the BET inhibitor in Ewing sarcoma cells [75].

Several studies implicate the IGF system in radioresistance. It is well established that ionizing radiation activates tyrosine kinase receptors involved in DNA damage response, including the IGF1R, as in fact targeting the IGF1R enhances radiosensitivity of different cancer cell lines [76,77]. A study conducted in murine glioma stem cells indicated that exposure to radiation increases IGF1 secretion, induces gradual increase in IGF1R expression, a decrease in phosphorylated Akt, activation of FoxO3a with consequent reduced proliferation, enhanced self-renewal through FoxO3 target genes and, ultimately, radioresistance [78]. Exposure of nasopharyngeal carcinoma cells to ionizing radiation boosted the expression of both phosphorylated IGF1R and γH2AX, a double-strand DNA breaks marker. Interestingly, combinatorial treatment with ionizing radiation and the anti-IGF1R agent linsitinib increased radiosensitivity by evoking G2-M cell cycle delay and enhanced apoptosis as compared to single treatments [79]. However, the molecular mechanisms underlying IGF1R-mediated radioresistance are cell context-dependent. In colorectal adenocarcinoma cells, IGF1R expression was associated with γ-irradiation resistance via transcriptional up-regulation of genes involved in DNA repair, including MSH4, RAD51 and BRCA2, rather than double strand breaks repair mechanisms [19].

Compensatory activation of various components of the IGF system often occurs in response to target therapies. In Ewing sarcoma, transcriptional up-regulation and autocrine activation of the IGF2/IR-A axis represents a major mechanism of resistance to anti-IGF1R agents [80]. Accordingly, dual IGF1R/IR inhibitors have been developed and their efficacy has been proved in different tumor types [57,81,82]. Over-activation of the IGF1R accounts for resistance to EGFR tyrosine kinase inhibitors. Interestingly, in non-small cell lung cancer cells, resistance to EGFR inhibitors was attenuated upon incubation with EGFR tyrosine kinase and IGF1R pathway inhibitors, which synergistically induce apoptosis by blocking Akt phosphorylation and inducing the expression of FoXO-regulated pro-apoptotic genes [83]. More recently, experiments conducted in Ewing sarcoma cells have demonstrated that IGF1R upregulation promotes resistance to CDK4/6 inhibitors suggesting that dual targeting of CDK4/6 and IGF1R may represent a synergistic combination with potential clinical implications for therapy in this disease [84]. Alterations in IGFBP2 expression are associated with resistance to both anti-IGF1R agents and dasatinib in rhabdomyosarcoma [85] and non-small cell lung cancer cells [86], respectively. Particularly, loss of IGFBP2 is associated with resistance to anti-IGF1R treatment due to hyperactivation of IGF signaling in rhabdomyosarcoma [85], while overexpression of IGFBP2 drives dasatinib resistance through activation of FAK in non-small cell lung cancer cells [86].

Metabolic reprogramming is a hallmark of cancer. Tumor cells metabolize glucose through aerobic glycolysis, rather than oxidative phosphorylation. This metabolic change determines an enhanced need of glucose uptake for ATP synthesis and generation of those metabolic intermediates necessary for biosynthesis of nucleotides, lipids and protein supporting cell proliferation [87,88]. Cancer cells also display metabolic flexibility, which allows the switch from glycolysis to oxidative phosphorylation and vice versa, supporting a role of mitochondria in cancer progression [89]. Experimental evidences have demonstrated a strong connection between the IGF system and metabolic reprogramming in cancer. IGF1R activation enhances glucose consumption, lactate and ATP production through the Akt pathway and consequent upregulation of GLUT1, a glucose transporter [90,91]. A study conducted by Vella and colleagues demonstrated that the IGF2/insulin/IR-A/PI3K/MAPK axis contributes substantially to energetic metabolic phenotype of breast cancer MCF-7 cells by increasing glycolytic activity, mitochondrial functions and cell bioenergetics [88]. In particular, IGF2 overexpression determined enhanced transforming capability of MCF7 cells compared to control cells as well as increased glucose consumption, increased lactate production, increased mRNA expression of glucose and lactate transporters and glycolytic enzymes. Significantly, the more aggressive phenotype was associated with increased mRNA expression of genes involved in mitochondrial biogenesis, fusion and activity, increased ATP production and enhanced glycolysis. Similarly, in breast cancer cells, IGF1 stimulates mitochondrial homeostasis by increasing oxidative phosphorylation to produce ATP required for proliferation. Particularly, IGF1 stimulates mitochondrial biogenesis and autophagy (mitophagy) through the PI3K pathway and induction of PGC-1β expression and PRC transcriptional activators, which support the transcription of mitochondrial genes and maintain mitochondrial morphology and mass, and induction of BNIP3, a major mediator of mitochondrial turnover [92].

## 4. Novel Regulators of the IGF System and Their Impact in Cancer

IGF inhibitors have failed in multiple clinical trials in part because of the complexity of IGF signaling [57,58]. Thus, the identification and characterization of novel regulators and binding partners of the IGF1R represents an emerging areas of cancer research. This section will highlight novel relevant interactions with the IGF axis, defining molecular mechanisms and their impact on tumor cells malignancy. Particularly, we will focus on post-transcriptional regulators and functional protein partners of IGF components.

### 4.1. Post-Transcriptional Regulators

Post-transcriptional regulators of gene expression dictate the entire RNA life cycle from alternative splicing, to nuclear export, transcript storage, stabilization and degradation with relevant implications in cancer (for a review [93]). These factors include non-coding RNAs, RNA-binding and RNA-editing proteins. Among those, the best characterized factors include microRNAs (miRNAs) and long non-coding RNAs (lncRNAs). As nicely previously reviewed [94,95], a large variety of miRNAs and lncRNAs regulate the IGF axis in tumor cells with functional implications in various stages of tumor malignancy. In addition, complex interactions among miRNAs and lncRNAs might also regulate various members of the IGF axis. A recent report indicates that the lncRNA NR2F1-AS1 promotes breast cancer angiogenesis via sponging miRNA 338-3p, thereby enhancing IGF1 expression and activating IGF1R signaling [96].

#### 4.1.1. Circular RNAs

**Figure 1:**
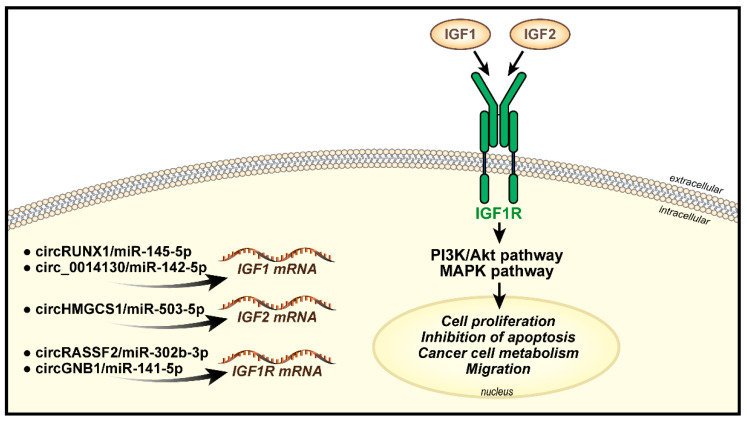
Schematic representation of circular RNAs governing IGF system in cancer. Circular RNAs sponge miRNAs binding to *IGF1*, *IGF2* and *IGF1R* mRNAs, thus promoting transcripts translation. IGFs/IGF1R interaction promotes receptor phosphorylation and downstream activation of the PI3K/Akt or MAPK pathways, which transmit signals to the nucleus and activate biological responses critical for neoplastic transformation.

Several studies have demonstrated the functional interaction between the IGF axis and circular RNAs (circRNAs) in cancer. circRNAs are a large class of single-stranded non-coding RNAs deriving from a non-canonical alternative splicing called “backsplicing”, which generates a covalent link between the 3′ and 5′ ends. circRNAs generally work by titrating miRNAs binding to mRNAs, thus influencing transcript stability and translation with implications in cancer [97]. circRNAs are aberrantly expressed in different tumor types and have been associated with both tumor initiation and progression in solid and hematologic malignancies [98,99,100]. In laryngeal squamous cell carcinoma, abnormal expression of circRNAs strongly correlates with malignant behavior. circRASSF2 displays upregulation in tumor tissues compared to normal controls as well as in cancer cell lines as compared to non-transformed cells. It enhances colony formation and migration while inhibiting apoptosis [101]. Interestingly, circRASSF2 regulates laryngeal squamous-cell carcinoma cells malignancy by sponging miR-302b-3p and enhancing the expression of IGF1R [101]. Similarly, circGNB1 sponges miR-141-5p and facilitates triple-negative breast cancer progression by upregulating the IGF1R [102]. A circRNA derived from backsplicing of the IGF1R itself, named circIGF1R, is detectable in hepatocellular carcinoma clinical specimens and promotes cell proliferation by activating the PI3K/Akt pathway [103]. Among other major components of the IGF axis, IGF1 is upregulated by the action of circRUNX1 on miR-145-5p or circ_0014130 on miR-142-5p, in colorectal [104] or nonsmall cell lung cancer [105], respectively, with substantial effects on cell proliferation, cell cycle progression, cell migration and inhibition of apoptosis. Recently published data support the impact of circRNA/IGF2 interaction in modulating cancer cell metabolism [106]. In hepatoblastoma cells, circHMGCS1 exerts its oncogenic role by sponging the tumor suppressing action of miR-503-5p, thus upregulating IGF2 and the PI3K-Akt signaling pathway [106]. In that cancer model, the circRNA/IGF2/Akt axis induced increased expression of the mRNA and protein for glutaminase, an enzyme that converts glutamine to glutamate, which is a major carbon source for ATP production in tumor cells [106,107]. Please refer to Figure 1 for a schematic representation of circRNAs controlling IGF system components in cancer.

#### 4.1.2. RNA-Binding Proteins: IGF2BPs Family

RNA-binding proteins (RBPs) account for 7.5% of the proteome [108], and each RBP binds hundreds of both coding and non-coding RNAs, thus playing a role in several physiological cellular processes. Accordingly, dysregulation of RBPs is frequently associated with pathological conditions including cancer [93]. RBPs mainly act in cytoplasmic complexes, called ribonucleoprotein (RNP) granules, which include mRNAs, RBPs, ribosome subunits and other proteins, devoted to RNA storage, stability, localization and degradation [93]. Different families of RBPs are dysregulated in cancer [109] such as the family of the insulin-like growth factor 2 mRNA binding proteins (IGF2BPs), which includes the paralogues IGF2BP1, IGF2BP2 and IGF2BP3 and plays an oncogenic role in different tumor models.

**Figure 2:**
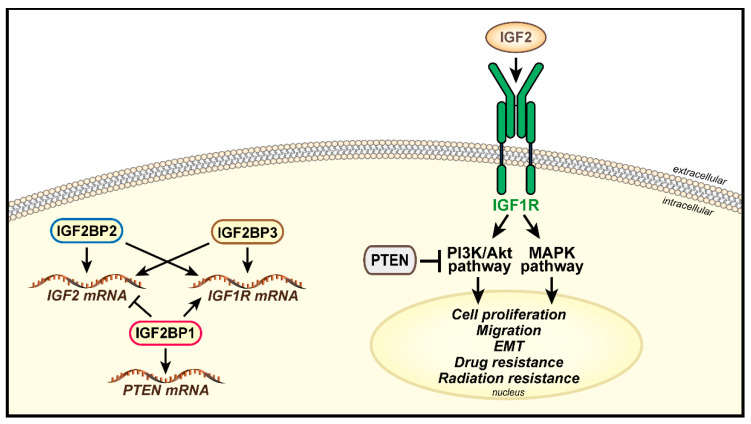
Cartoon depicting RNA-binding proteins IGF2BP1, IGF2BP2 and IGF2BP3 activity on the IGF system in cancer. IGF2BPs differentially regulate *IGF2*, *IGF1R* or *PTEN* transcripts. IGF2BP1 inhibits *IGF2* mRNA but sustains *IGF1R* and *PTEN* mRNAs translation. IGF2BP2 and IGF2BP3 favor *IGF2* and *IGF1R* mRNAs translation. The functional interaction between IGF2/IGF1R/PTEN proteins is shown. IGF2 binding to the IGF1R activates downstream pathways such as the PI3K/Akt, which is negatively regulated by PTEN, and MAPK.

IGF2BPs were originally identified as major negative post-transcriptional regulators of IGF2 during embryonic late development [110]. IGF2BPs expression is lost in adult tissues while de novo expression occurs in many human tumors where IGF2BPs associate with poor outcome and cell malignancy (for a comprehensive review [111,112,113,114]). IGF2BPs regulate the IGF1R at post-transcriptional level. As shown in hepatocellular carcinoma and Ewing sarcoma cells, knockdown of IGF2BPs is associated with downregulation of IGF1R mRNA and protein and consequent inhibition of in vitro cell viability, proliferation, clonogenicity and migration [81,115,116]. Interestingly, in Ewing sarcoma cell lines, IGF2BP3-mediated IGF1R loss is compensated by the activation of an IR-A/IGF2 autocrine loop [81], which determines higher sensitivity to the dual IGF1R/IR inhibitor OSI-906 [81]. However, it is important to mention that IGF2BPs display peculiarities in their mechanisms of action, which is often dependent on cell context [111,114]. Notably, IGF2BP1 inhibits *IGF2* mRNA translation, while opposite effect is elicited by IGF2BP2 and IGF2BP3 [82,117,118,119,120,121,122,123]. Accordingly, in breast cancer cells, IGF2BP1 knockdown determined increased mRNA expression for *IGF2* associated with inhibition of cell proliferation [117]. Collectively, these data support the notion that IGF2 might not be the major mediator of IGF2BP1-modulated oncogenic effects in cancer. Relevant to IGF signaling, IGF2BP1 binds a codon-comprising fragment of the *PTEN* ORF, thus preventing *PTEN* mRNA decay. In tumor cells, the IGF2BP1-PTEN axis antagonizes the activation of Akt and HSP27, which modulates the actin cytoskeleton and influences cell migration [124]. Accordingly, the IGF2BP1/PTEN/Akt/HSP27 axis drives cell migration in different tumor types, including hepatocellular carcinoma, by regulating directionality of cell migration through lamellipodia formation and cell polarization [124,125]. IGF2BP2 binds the *IGF2* 5-’untranslated region (UTR)**,** thus favoring *IGF2* translation [119] and promoting tumorigenesis and cell proliferation via the PI3K/Akt pathway [118,120,121]. In glioblastoma cells, the IGF2BP2/IGF2/Akt axis promoted cell motility and EMT, as demonstrated by increased mRNA and protein expression of vimentin and N-cadherin. In addition, IGF2BP2 overexpression mediates resistance to temozolomide [126,127]. Interestingly, the ability of IGF2BP2 to promote proliferation and IGF2 expression depends on mTOR-mediated phosphorylation suggesting a mechanism of positive feedback loop [118]. Similarly, IGF2BP3 binds the *IGF2* 3′-UTR, thus promoting its translation, increasing activation of IGF signaling and cell proliferation in leukemia [122], thyroid cancer [82] and glioma [123]. In thyroid cancer cells, over expression of the IGF2BP3/IGF2 axis correlates with enhanced activation of the Akt and ERK pathways, increased cell proliferation and sensitivity to the dual inhibitor OSI-906 [82]. In addition, the IGF2BP3/IGF2 axis promotes resistance to ionizing radiation. In chronic myeloid leukemia cells IGF2BP3 depletion determined a reduction of IGF2 production and increased susceptibility to ionizing radiation as measured by apoptosis, effect that was partially reversed by treatment with recombinant IGF2 [128]. We have very recently demonstrated that IGF2BP3 controls the expression of the chemokine receptor CXCR4 through post-transcriptional regulation of its functional partner CD164 [129], thus promoting the motility of Ewing sarcoma cells toward the CXCR4-ligand CXCL12 under hypoxia conditions [129]. Interestingly, a crosstalk between CXCR4 and IGF1R in cancer cells has been described. Particularly, in breast cancer, cells IGF1R and CXCR4 directly interact at the cell membrane [130], and this interaction allows IGF1 to promote cell migration through transactivation of CXCR4 [130]. Overall, these results suggest a putative role of IGF2BP3/IGF axis in the regulation of CXCR4-mediated signaling pathway, although this hypothesis remains speculative for now. IGF2BPs activity on the IGF system in cancer is summarized in Figure 2.

#### 4.1.3. ADAR and A-to-I RNA Editing

**Figure 3:**
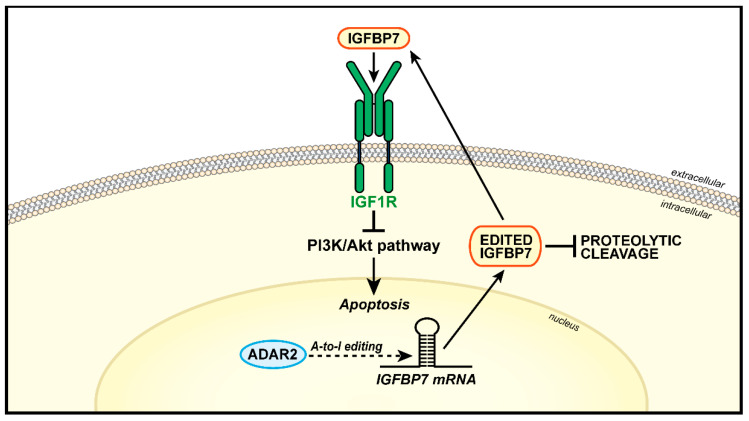
Representation of ADAR2-mediated RNA editing and its action in regulating the IGF system in cancer. A-to-I RNA editing of *IGFBP7* mRNA guided by ADAR2 leads to increased production of a full-length edited IGFBP7 protein, which is resistant to proteolysis. Secreted IGFBP7 binds IGF1R, thus inhibiting the PI3K/Akt pathway and inducing apoptosis.

The adenosine deaminases acting on RNA (ADAR) proteins are responsible for selective deamination of adenosines (A) to inosines (I) in double-stranded RNA molecules, leading to major changes in target RNAs sequences. The edited codon generates a different amino acid as compared to its genomically encoded original, which can affect protein stability, localization, and function [131]. Bioinformatics screens have identified *IGFBP7* mRNA as a putative target of ADARs [132]. IGFBP7 is a secreted factor that suppresses cancer cells protein synthesis, growth and survival by competing with IGF1 binding to the IGF1R, thereby preventing its activation [133]. A-to-I editing of *IGFBP7* generates different isoforms, which are differentially susceptible to proteolytic cleavage, an event that profoundly impacts the biological activity of this protein [131]. Full-length IGFBP7, but not truncated isoforms, binds the IGF1R and inhibits downstream signaling [133]. As recently shown in esophageal squamous carcinoma cells, RNA editing mediated by ADAR2 protects full length IGFBP7 from proteolysis, thus inducing apoptosis through the inhibition of Akt activation and BAD phosphorylation [134]. Collectively, these results suggest an oncosuppressive role for ADAR2 in this tumor type [134]. A schematic representation of ADAR2-mediated RNA editing on IGFBP7 and IGF axis in cancer is summarized in Figure 3.

### 4.2. Functional Protein Partners

In recent years, novel proteins affecting the activity of the IGF1R and IR in cancer have been identified. In the next paragraphs, we will discuss the crosstalk between IGF1R/IR and some novel partners and the role that these interactions play in regulating cancer initiation and progression.

#### 4.2.1. DDR1

Several studies demonstrated that the discoidin domain receptor 1 (DDR1) is a signaling partner of the IGF1R and IR [135,136]. DDR1 belongs to a family of membrane receptor tyrosine-kinases, including DDR1 and DDR2, that bind to and are activated by various forms of collagen [137,138,139]. Structurally, DDRs are characterized by an extracellular N-terminal discoidin domain, which binds collagens, a juxtamembrane domain, and a catalytic tyrosine kinase domain, which undergoes phosphorylation and activates downstream signaling [140]. In cancer, DDRs are over expressed in different tumor types and play a role in cancer progression. Published data suggest that DDR2 functionally interacts with the IR but the detail of this interaction is not fully defined [141]. On the contrary, the functional crosstalk between DDR1 and the IGF1R or IR has been better characterized. DDR1 associates with IGF1R or IR-A in response to IGF1 or insulin/IGF2, respectively, enhancing IGF1R/IR-A expression levels, Akt and MAPK activation and downstream biological responses [142,143,144,145]. Accordingly, DDR1 depletion inhibits IGF1R- or IR-A-elicited proliferation, migration and colony formation after cognate ligand stimulation [142,144]. More recent data have demonstrated that DDR1 is upregulated in bladder cancer tissues and cell lines, where it functionally interacts with both the IGF1R and the IR and modulates ligand-evoked bladder cancer cell motility [146]. DDR1 complexes with Pyk2, non-muscle myosin IIA, the IGF1R or IR in ligands-dependent fashion, linking the IGFIR and IR-A to the regulation of F-actin cytoskeleton [146].

**Figure 4:**
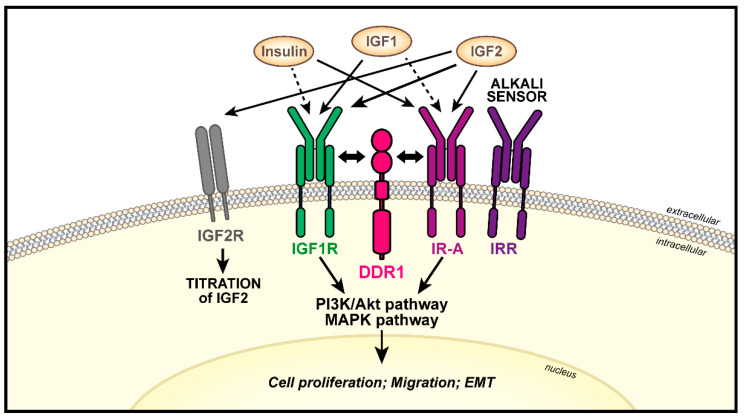
Schematic representation of the functional crosstalk between DDR1 and the IGF system in cancer. High or low ligand/receptor affinities are represented by continuous or dot arrows, respectively. DDR1 directly interacts with the IGF1R and IR-A thereby modulating the activation of IGF1R/IR-A downstream signaling pathways leading to transformation. As depicted, the IGF system includes two additional receptors, the IGF2R and IRR. However, whether these receptors may functionally interact with DDR1 has not been reported.

DDR1 crosstalk with the IGF axis can also modulate cell differentiation [145]. In undifferentiated thyroid cancer cells, DDR1 downregulation inhibits the IGF2/IR-A signaling pathway and increases the expression of differentiation markers, such as TSH and TPO, and decrease of EMT and stemness markers, like Nanog, ABCG2 and vimentin [145]. On the contrary, IGF1R depletion impairs collagen-dependent phosphorylation of DDR1, further pointing out the reciprocity of this functional crosstalk [142,144]. Please refer to Figure 4 for a schematic representation of the interaction between DDR1 and the IGF system in cancer.

#### 4.2.2. Decorin

**Figure 5:**
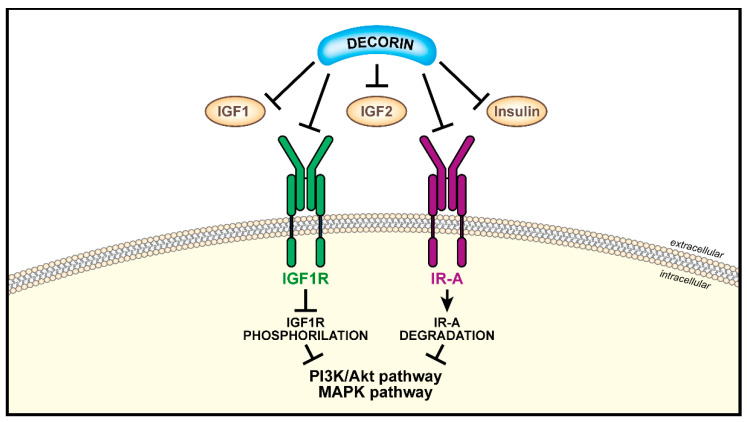
Schematic representation of antagonistic functional effects driven by decorin on the IGF system. In tumor stroma, decorin binds the ligands IGF1, IGF2 and insulin as well as the receptors IGF1R and IR-A. Decorin inhibits ligand-mediated IGF1R phosphorylation while it enhances IGF-2-mediated IR-A protein degradation. Negative decorin action on downstream pathways is reported.

Decorin, the prototype member of the small leucine-rich proteoglycans [147,148,149] is a key component of tumor stroma and acts as a tumor suppressor in cancer by down-regulating the activity of several tyrosine-kinase receptors [150], including the IGF1R and IR-A [151,152]. Decorin regulates the IGF system at multiple levels with mechanisms that substantially differ between physiological and pathological cell models [153]. As shown in bladder cancer cells, decorin binds with similar affinities both the IGF1R and IGF1 at distinct sites and inhibits IGF1-mediated IGF1R phosphorylation, without affecting IGF1R protein levels [151]. On the contrary, decorin enhances IGF1-evoked IRS-1 degradation and inhibits Akt and MAPK activation, thus blunting the ability of the IGF1R to promote ligand-evoked bladder cancer cells migration and invasion [151]. Decorin also binds with high affinity the IR-A and its cognate ligands insulin and IGF2 [152]. However, decorin does not affect ligand-dependent phosphorylation of the IR-A but it enhances instead IGF2-mediated IR-A protein degradation and inhibits IGF2-dependent activation of Akt and cell growth [152]. Collectively, these results suggest that decorin may act as a natural antagonist of the IGF1R and IR-A in bladder and other types of cancer where these receptors might play a critical role. Figure 5 schematizes the effects driven by decorin on the IGF system in cancer.

#### 4.2.3. E-Cadherin

**Figure 6:**
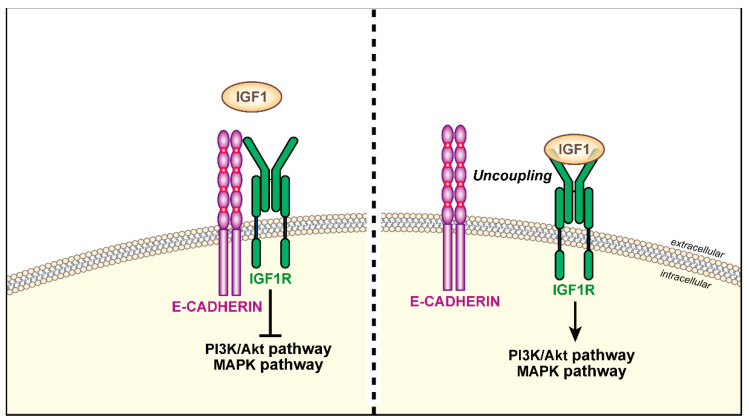
Cartoon showing E-cadherin functional interaction with the IGF1R in cancer. On the left side, E-cadherin interacts with the IGF1R at the cell membrane leading to downregulation of IGF-dependent signaling pathways. On the right side, the E-cadherin/IGF1R interaction is disrupted by IGF1 stimulation with consequent activation of downstream signaling pathways.

E-cadherin belongs to a superfamily of calcium-dependent adhesion molecules with a critical role in cell adhesion [154]. Changes in E-cadherin level is one of the hallmarks of EMT, a crucial program in the regulation of cell motility and invasion, metastasis, chemoresistance and stemness [155,156]. Accordingly, during EMT, cells undergo upregulation of N-cadherin and loss of E-cadherin (for a review please consider [155]). As previously mentioned, the activation of the IGF system in cancer is strongly associated with EMT [66,67,68,69]. Intriguingly, data from the literature indicate that E-cadherin represents a regulator of IGF1R and IR action. In breast cancer cells, E-cadherin downregulation hyperactivates IGF1R/IR signaling, thus enhancing sensitivity of breast cancer cells to IGF1 stimulation and Akt or ERK phosphorylation [4,157]. At functional levels, E-cadherin depletion determines increased cell cycle progression and enhanced sensitivity to IGF1R/IR-targeted therapy [157]. Mechanistically, E-cadherin and IGF1R colocalize to adherens junctions, and this interaction is significantly decreased after IGF1 stimulation indicating that the E-cadherin/IGF1R interaction is disrupted for proper IGF1R function [157]. Similar results were also obtained in colon cancer cell lines: IGF1 binding to IGF1R disrupts the E-cadherin/IGF1R interaction on cell surface with subsequent repositioning of the IGF1R and E-cadherin from cell-to-cell to focal contacts, thereby leading to enhanced cell migration [4,158]. A schematic representation of the functional interaction between E-cadherin and IGF1R is shown in Figure 6.

## 5. Concluding Remarks

In spite of decades of research in the field, many unanswered questions still remain about the mechanisms regulating IGF system-evoked biological responses. Aberrant IGF bioactivity modulates critical cellular processes including EMT, resistance to chemo and targeted therapies, epigenetic drugs, glycolytic activity and mitochondrial functions. The multiplicity of dysregulated responses elicited by this system reflects the complex scenario of regulators acting on the components of IGF system. The vast majority of the information obtained to date indicate the regulatory functions of multiple post-transcriptional and functional protein partners, with either agonistic or antagonistic activity, which integrate the molecular signaling pathways mediated by the IGF axis. A better understanding of these regulatory networks in cancer might contribute to discovering novel approaches to control cancer development and progression.
